# Indoor Trajectory Tracking Scheme Based on Delaunay Triangulation and Heuristic Information in Wireless Sensor Networks

**DOI:** 10.3390/s17061275

**Published:** 2017-06-02

**Authors:** Junping Qin, Shiwen Sun, Qingxu Deng, Limin Liu, Yonghong Tian

**Affiliations:** 1 School of Computer Science and Engineering, Northeastern University, Shenyang 110819, China; qinjunping30999@imut.edu.cn; 2 College of Information Engineering, Inner Mongolia University of Technology, Hohhot 010080, China; supersunshiwen@Gmail.com (S.S.); liulimin@imut.edu.cn (L.L.); tyh@imut.edu.cn (Y.T.)

**Keywords:** wireless sensor network, *RSSI*, Delaunay triangulation, heuristic information, trajectory tracking, dynamic time warping

## Abstract

Object tracking and detection is one of the most significant research areas for wireless sensor networks. Existing indoor trajectory tracking schemes in wireless sensor networks are based on continuous localization and moving object data mining. Indoor trajectory tracking based on the received signal strength indicator (*RSSI*) has received increased attention because it has low cost and requires no special infrastructure. However, *RSSI* tracking introduces uncertainty because of the inaccuracies of measurement instruments and the irregularities (unstable, multipath, diffraction) of wireless signal transmissions in indoor environments. Heuristic information includes some key factors for trajectory tracking procedures. This paper proposes a novel trajectory tracking scheme based on Delaunay triangulation and heuristic information (TTDH). In this scheme, the entire field is divided into a series of triangular regions. The common side of adjacent triangular regions is regarded as a regional boundary. Our scheme detects heuristic information related to a moving object’s trajectory, including boundaries and triangular regions. Then, the trajectory is formed by means of a dynamic time-warping position-fingerprint-matching algorithm with heuristic information constraints. Field experiments show that the average error distance of our scheme is less than 1.5 m, and that error does not accumulate among the regions.

## 1. Introduction

Currently, many indoor monitoring systems are based on wireless sensor networks (WSNs), such as safety monitoring systems in coal mines, disaster early warning systems in subway tunnel construction and indoor navigation systems. Indoor monitoring applications have undergone rapid development.

The position and moving trajectory of a monitored object form the most basic information for location based services. Knowing the position and moving trajectory of a miner is the most crucial factor in mine disaster rescue operations. Similarly, an early warning signal can be triggered by means of the relationship between the relative positions of vehicles and workers in a subway tunnel site. It is meaningless for wireless sensor networks to collect data without positional information [1].

Based on the principles for forming trajectory, trajectory tracking methods mainly include the following: trajectory tracking based on localization, trajectory tracking based on acceleration measurements, and trajectory tracking based on video recognition.

Trajectory tracking based on localization consists of two phases. First, a series of positions of an object are determined by sharing data between reference nodes and unknown nodes. Then, the trajectory of the moving object is created using moving-object data-mining techniques [2].

Outdoor localization and trajectory tracking are based on satellite localization systems, for example, Global Positioning Systems (GPSs), have been widely used [3]. However, GPSs are often ineffective indoors because of signal propagation limitations in indoor environments [4]. A universal formal description of localization for wireless sensor networks was proposed from the standpoint of mathematical modeling that covers the main ranging measurement and regional estimation methods [5]. The accuracy of trajectory tracking is largely determined by localization accuracy. In recent years, researchers have paid more attention to indoor localization based on the received signal strength indicator (*RSSI*), which represents the received power level at a receiver, because this approach requires no special infrastructure and has no Line of Sight (LOS) limitations [6]. Indoor localization based on fingerprinting was first proposed by Microsoft Research (Redmond, Washington, USA) with the development of the RADAR system [7]. This localization system uses a fingerprint, which is a vector formed by the *RSSI*s from multiple adjacent reference nodes. The localization method based on the position fingerprint consists of two phases: in the offline phase, an area is divided into a series of grid units and the *RSSI* measurements are collected at each intersection. To avoid the impact of different device orientations, the *RSSI* information is collected for each of the four directions (north, south, east, or west). Each combination of position and orientation (i.e., an (x, y, d) tuple), (tuple, *RSSI* vector mean) is stored in a fingerprint map table for use in the online phase. The mean is the average value of multiple samples. In the online phase, the *RSSI* information of a moving object will be detected and recorded. Then, the received *RSSI* vector will be compared with the fingerprint map table to determine the location of the moving object.

Multipath propagation is a main feature of wireless communication [7]. It is a kind of communication phenomenon that the wireless signal transmitted from a transmitter antenna arrives at a receiver antenna along two or more paths. Reflections, refractions and scattering are generated during the propagation process of wireless signal. Therefore, it is possible to receive the multiple same source electromagnetic waves at an arbitrary position, which is multipath propagation. The received signal is a synthesis of the direct path wave and multiple other waves. The amplitude change of the received signal is known as multipath fading.

*RSSI*-based indoor localization is particularly challenging for the following three reasons [6]. First, it is difficult to obtain accurate *RSSI* measurements. According to the results of experiments in [8], the standard deviation of *RSSI* measurements collected from a stationary node in one minute can be up to 5 dBm. Second, distance estimation based on *RSSI* measurements can easily be affected by multipath and diffraction effects, which contribute to most of the estimation errors in current indoor ranging measurement localization systems. Third, manufacturing variations among different wireless devices may also result in RSSI measurement discrepancy.

An in-depth analysis on the relationship between *RSSI* and LQI (link quality indicator) is proposed in [9]. LQI represents the energy and quality of the received data frames. Its value is based on the signal strength and the detected SNR (Signal-to-Noise Ratio). LQI is calculated by the MAC (Media Access Control) layer and uploaded to the upper layer. LQI is related to the probability of the correctly receiving data frames.

*RSSI* and LQI can be read from the built-in indicator in the CC2530 chip. Experiments [10] show that the level of LQI determines the quality of *RSSI* value. For a stationary node, the better the LQI is, the more stable the *RSSI* is; otherwise, *RSSI* fluctuates markedly. *RSSI* is directly related to the transmission distance, and LQI is directly related to signal modulation and interference.

Compared to *RSSI*, channel state information (CSI) [8] yields more accurate values from the physical layer; CSI describes the amplitude and phase, two properties that are characteristic of each subcarrier in the frequency domain. CSI information is more temporally stable than *RSSI*. More importantly, the CSI values of multi-subcarriers reasonably reflect the multipath effects. Based on these advantages, a CSI-based indoor localization scheme can achieve good performance. However, only orthogonal frequency division multiplexing (OFDM)-based wireless systems can extract the CSI because they use multiple subcarriers for data transmission; other modulation schemes such as direct sequence spread spectrum cannot provide this value.

Trajectory tracking methods that make use of real-time acceleration information to track an object are described in [11]. A future position of an object can be inferred from knowledge of its present position, speed and real-time acceleration information. To measure acceleration and speed in real time, acceleration sensors and gyroscopes are used in robots or aircrafts. However, without real-time calibration in indoor environments, this method results in large accumulated errors.

Trajectory tracking based on consecutive object recognition in videos has been one of the most active research topics in recent decades, particularly in the field of machine vision [12]. Visual tracking remains a challenging problem due to many factors, e.g., shifting viewpoint, signal noise, background clutter, illumination changes, and object deformation due to movement and image overlapping. A tracking mechanism was proposed in [13] that combined a structural regional hierarchy method to represent an object with a novel graph-matching algorithm. This algorithm has robust performance due to the hierarchical nature of its object representation. However, this method requires special video capture infrastructure; consequently, it is impractical in WSNs.

The continuous position fingerprint of a single object over time must correspond to its adjacent position in space. This spatiotemporal correlation can be regarded as a constraint in the trajectory tracking procedure. Some heuristic information can be mined in conjunction with the deployment of reference nodes. Existing indoor trajectory tracking schemes for WSNs are mainly based on continuous localization methods and cannot make use of heuristic information of an *RSSI* time series within a certain temporal and spatial range. The heuristic information of an *RSSI* time series includes some key factors for the trajectory tracking procedure. This study aims to design a novel trajectory tracking scheme that mines heuristic information to improve the accuracy of trajectory tracking. In this paper, the position of a moving node on the boundary is determined according to the change tendency of *RSSI*, and the moving trajectory is determined according to the *RSSI* time series.

In summary, the main contributions of this paper are as follows:

1. A novel indoor trajectory tracking scheme for WSNs is proposed based on Delaunay triangulation and heuristic information (TTDH). TTDH combines range free localization algorithms with position fingerprint localization algorithms. The error caused by uncertainty and fluctuation of *RSSI* can be effectively restrained in TTDH.
2. Algorithms that extract heuristic information from *RSSI* time series are designed. Heuristic information includes some key factors for the trajectory tracking procedure. The principle of heuristic information is strictly proven mathematically. The temporal information and spatial information in WSNs are fully utilized to mine the heuristic information.
3. The moving trajectory of an object is formed by means of a dynamic time-warping-position-fingerprint-matching algorithm with heuristic information constraints. TTDH has a good robustness, and that error does not accumulate among the regions. Field experiments show that the average error distance of the tracked trajectory is less than 1.5 m.

The rest of this paper is organized as follows. Section 2 first provides some related background information. Section 3 presents the TTDH scheme and the algorithm design. In Section 4, we provide the results of experiments and a discussion. Finally, we present conclusions in Section 5.

## 2. Related Work

Trajectory tracking is a continuous localization process. A conventional trajectory tracking method first locates an object and then forms a trajectory using moving-object data-mining techniques. In contrast, using heuristic information, we consider trajectory tracking and localization as a whole. This section introduces the basic theory of Delaunay triangulation. We propose and prove the peak phenomenon on the boundary. Then, the approximate point in the triangulation (APIT) test principle is introduced to determine sequential triangular regions.

**Figure 1:**
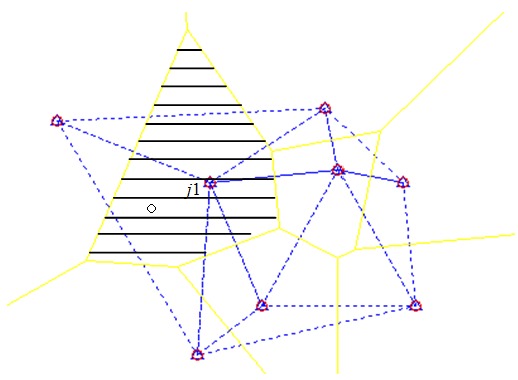
Voronoi diagram and Delaunay triangulation.

After the nodes are deployed at random, using a Voronoi diagram, a two-dimensional localization plane is partitioned into Voronoi cells (see Figure 1). A Voronoi cell is a region enclosed by real lines. A Voronoi cell could be either half-open or closed. The black symbol ∘ represents an unknown node and the red symbol ∘ represents a reference node in the center of a Voronoi cell. An unknown node must belong to a Voronoi cell whose center is the nearest among all reference nodes. That is,

(1) $$\begin{array}{c} 0<L_{i-j1}\leq L_{i-j2}\cdots \leq L_{i-jk}\leq R, \end{array}$$

where *R* represents the communication radius between a reference node and an unknown node, j1,j2,…,jk represents a sequence of reference nodes that can communicate directly with the unknown node, *k* represents the number of these reference nodes, and $L_{i-jk}$ represents the distance between the unknown node *i* and the reference node jk. The unknown node ∘ belongs to the shaded region named Voronoi cell j1 in Figure 1.

Delaunay triangulation is the dual graph of a Voronoi diagram. As shown in Figure 1, each triangle is enclosed by dotted lines and each side of the triangle links two reference nodes. Delaunay triangulation is an optimized triangulation in the sense of “maximized minimal angle”. Many applications in WSNs are based on Delaunay triangulation. For example, Wu et al. [14] used Delaunay triangulation to find the largest free space within a WSN that could hold the next deployment target. Constrained Delaunay triangulation is widely used in Geographic Information System (GIS) applications [15] and is embedded in common GIS software (e.g., the ArcGIS software).

Calculation of a Delaunay triangulation requires global information, namely, the exact positions of all the reference nodes in the WSN. We perform this calculation task in the central computer (a personal computer). The resulting Delaunay triangulation scheme will be used consistently unless a reference node is moved.

The total number *n* of Delaunay triangular regions satisfies Euler’s formula in topology:

(2) $$\begin{array}{c} e=p-l+n, \end{array}$$

where *p* represents the total number of reference nodes, *l* represents the total number of sides, and Euler’s characteristic *e* is a constant. When the total number *p* of reference nodes is specified, *l* and *n* will vary because reference nodes are deployed differently.

## 3. Heuristic Information Detection and Constrained Fingerprint Matching Algorithm

As discussed above, *RSSI* measurements are easily affected by various factors. To improve the accuracy of localization and trajectory tracking, methods such as dimensionality reduction [16], multivariate statistics [17], and support vector machine [18,19] have been introduced into the fingerprint matching algorithm. Our scheme mines some heuristic information, which reveals the spatiotemporal correlation in conjunction with the process of trajectory tracking.

### 3.1. Peak Value Phenomenon on the Boundary

**Figure 2:**
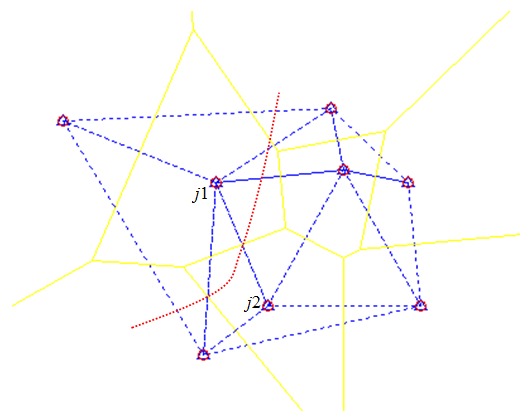
Heuristic information based on Delaunay triangulation.

Based on the excellent features of Delaunay triangulation, a common side is a line segment between the two closest reference nodes. Our scheme considers a common side as a boundary between two adjacent triangular regions. As an unknown node moves continuously, it will pass through every triangle along its moving trajectory; therefore, it will cross over the common side between adjacent triangles. As shown in Figure 2, the common side j1j2 must intersect with the moving trajectory. Consequently, the *RSSI* measurements received from the two reference nodes can be considered as a time series $R_{b}$:

(3) $$\begin{array}{c} R_{b}=\left(r_{b1},r_{b2},r_{b3},\ldots ,r_{bk},\ldots ,r_{bl}\right), \end{array}$$

(4) $$\begin{array}{c} r_{bk}=\left(r_{j1k}+r_{j2k}\right)/2. \end{array}$$

For example, assume that j1j2 is a common side that represents a boundary. $R_{b}$ represents the *RSSI* time series related to boundary j1j2. *b* is the *b*th boundary, *l* is the length of boundary time series $R_{b}$, and $r_{j1k}$ and $r_{j2k}$ represent *RSSI* measurements received separately from j1 and j2 at nearly the same time. The time series element $r_{bk}$ is the mean of $r_{j1k}$ and $r_{j2k}$, and *k* is the *k*th element.

As an unknown node gradually approaches, intersects, and moves away from the common side, $R_{b}$ will increase during the approach phase and decrease during the departing phase. These changes tend to coincide with the wireless signal transmission model.

**Theorem** **1.** Provided there is no noise, the maximal value of $R_{b}$ will be located at the intersection between boundary j1j2 and the moving trajectory.

**Figure 3:**
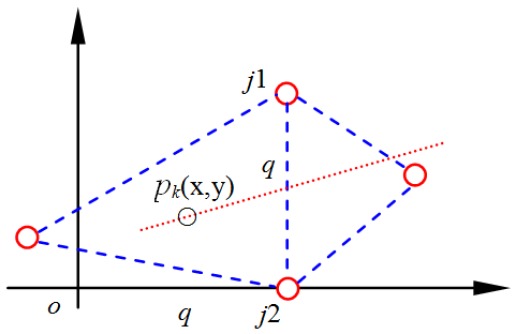
Relationship between boundary j1j2 and moving trajectory.

**Proof** **of** **Theorem** **1.** As shown in Figure 2, boundary j1j2 could be in an arbitrary direction. We first rotate and translate the coordinates to ensure that boundary j1j2 is vertical. As shown in Figure 3, ∘j2=j1j2=q. $P_{k}$ represents the *k*th position related to boundary j1j2 time series $R_{b}$. *x* and *y* are the position coordinates of the unknown node at this time.

For a radio channel, wireless signal propagation in an indoor environment is dominated by multipath, diffraction, and scattering of the radio waves caused by structures within the building [7]. There are several wireless signal transmission models, including the free-space propagation model, the Rayleigh fading model and the logarithmic shadowing model. The logarithmic shadowing model considers the factors mentioned above synthetically and is both simpler and more accurate than other models in indoor environments [20]. We adopt this model for further discussion. The relationship between the *RSSI* and propagation distance can be described as follows:

(5) $$\begin{array}{c} \frac{P(d_{0})}{P(d)}={\left(\frac{d}{d_{0}}\right)}^{\beta }, \end{array}$$

(6) $$\begin{array}{c} R(d)=10\mathrm{lg}P(d), \end{array}$$

(7) $$\begin{array}{c} R\left(d\right)=R\left(d_{0}\right)-10\beta \mathrm{lg}\left(d/d_{0}\right), \end{array}$$

where R(d) (dBm) represents the *RSSI* related to the propagation distance *d*. $R(d_{0})$ represents the *RSSI* at the reference node whose propagation distance is $d_{0}$. P(d) (mw) represents the mean received signal power related to the propagation distance *d*, and $P(d_{0})$ represents the mean received signal power at the reference node whose propagation distance is $d_{0}$. In general, $P(d_{0})$ takes the mean received signal power when $d_{0}=1$ m. The path attenuation factor, β, indicates the rate at which the path loss of the mean received signal power increases with distance. In theory, β will vary according to the environment. However, it is reasonable to assume that β is a constant within a small range. From Equation (7), it is evident that the *RSSI* will decrease as the propagation distance *d* increases when β satisfies certain conditions. We regard $r_{bk}$, which is related to the moving trajectory (see Figure 3), as a function of the point $p_{k}\left(x,y\right)$:

(8) $$\begin{array}{c} r_{bk}=2R\left(d_{0}\right)-10\beta \mathrm{lg}\left(\sqrt{{\left(q-x\right)}^{2}+{\left(q-y\right)}^{2}}\right)-10\beta \mathrm{lg}\left(\sqrt{{\left(q-x\right)}^{2}+y^{2}}\right), \end{array}$$

(9) $$\begin{array}{c} \frac{\partial (r_{bk})}{\partial x}=\frac{10}{2\ln10}\left[\frac{\beta (x-q)}{\left[{\left(q-x\right)}^{2}+{\left(q-y\right)}^{2}\right]}+\frac{\beta (x-q)}{\left[{\left(q-x\right)}^{2}+y^{2}\right]}\right]. \end{array}$$

As shown in Figure 3, *q* is the length of boundary j1j2. When x=q, $\frac{\partial (r_{bk})}{\partial (x)}=0$. In view of this real problem, $r_{bk}$ will achieve its maximal value only at the stationary point, and this stationary point must be on boundary j1j2.  ☐

The phenomenon illustrates a definite change tendency about $R_{b}$. The maximal value could occur at a small deviation relative to boundary j1j2 due to discrete *RSSI* measurement errors. We will discuss this problem further in Section 4.

Based on experimental data, a peak value phenomenon will occur with a high likelihood when the length of a boundary is below 50% *R*, where *R* represents the communication radius. Without loss of generality, the deployment of reference nodes can be eligible.

### 3.2. Delaunay Triangulation Inside/Outside Decision Based on APIT

The approximate point in the triangulation (APIT) [21,22] localization method is a typical range-free localization scheme and is widely used for position estimation in WSNs because of its robustness to irregular wireless transmission models and random node distributions. The APIT test is an approximate algorithm of the point in triangulation (PIT) method. We first introduce PIT.

**Figure 4:**
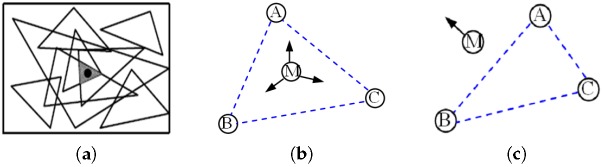
PIT (point in triangulation) principle: (**a**) Position estimation; (**b**) Inside; (**c**) Outside.

As shown in Figure 4a, many triangular regions are formed by reference nodes. PIT uses the change tendency of the *RSSI* to estimate the triangular region in which an unknown node is located. The relationship between an unknown node M and a triangular region ΔABC is that the node is either inside or outside the triangle. The reference nodes A, B, C are three vertices of the Delaunay triangle in which the unknown node M resides. In the first case, when the unknown node M is moved, the distances MA, MB, and MC impossibly increase or decrease simultaneously (see Figure 4b). In the other case, when the unknown node M is moved, at least in one direction, the distances MA, MB, and MC must definitely increase or decrease simultaneously (see Figure 4c). According to the monotonic relationship between the *RSSI* and propagation distance, if a direction exists such that the *RSSI* measurements received by the unknown node M from the three reference nodes increase or decrease simultaneously in this direction, then we can determine that the unknown node M is outside the triangle formed by the three reference nodes. Otherwise, M is inside the triangle. This is the PIT test. Essentially, PIT is an exhaustive approach that is infeasible in practice.

**Figure 5:**
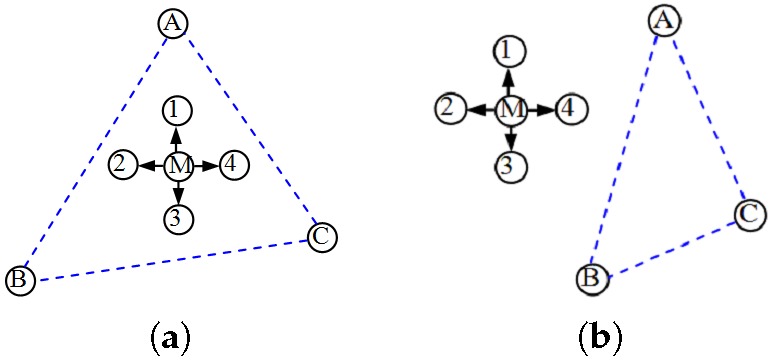
APIT (approximate point in the triangulation) principle: (**a**) Inside; (**b**) Outside.

Exchanging adjacent node information in APIT is regarded as node movement in PIT. As shown in Figure 5a, 1, 2, 3, and 4 are adjacent nodes of M. If no identical change (increase/decrease) occurs between M and each adjacent node, M is assumed to be inside the triangular region ΔABC. Otherwise, M is outside this triangular region. As an approximate substitute, experiments have confirmed that the error rate of APIT tests is relatively low (less than 14% in the worst case) when the node density (the average number of nodes per node radio area) is greater than 6 [21].

**Figure 6:**
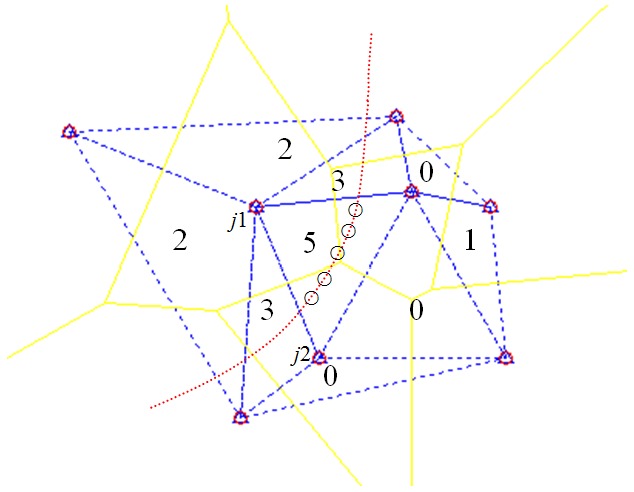
APIT based Delaunay triangulation inside decision.

A series of APIT tests are performed during the process of node movement. Unlike classic APIT methods, Delaunay triangulation is regarded as a triangular unit in our TTDH. A group of variables are introduced to aggregate the processed results, and each variable is initially set to 0. The number of variables is equal to *n* in Equation (2). If M is inside a triangular unit, the corresponding variable is incremented by 1. If M is outside a triangular unit, the corresponding variable maintains unchanged. As shown in Figure 6, the variables related to a triangular unit crossed by the trajectory are larger than the other variables. We can then determine which triangular units the unknown node has crossed. Compared with the APIT method, continuous aggregation can improve the accuracy rate of a triangular unit decision.

The numbers in triangular units are the accumulated results (see Figure 6). There is an error (number 1) related to a single APIT test, but it does not typically affect the precision of the entire decision (it can sometimes be affected; we will discuss this problem further in Section 4). It is evident from Figure 6 that these triangular units, related to the numbers 2, 3, 5, 3, and 2 from left to right, are considered triangular regions crossed by the trajectory.

After the sequential triangular regions have been detected, the common sides of adjacent triangular regions must be the boundaries intersected by the trajectory.

The pseudocode for the Delaunay triangulation inside/outside decision based on APIT is as Algorithm 1:
**Algorithm 1** Delaunay Triangulation Inside/Outside Decision Based on APITInput: Delaunay triangulation of an experiment site, the *RSSI* of an object node and its surrounding nodes; Output: the sequential triangular regions where the monitored object resides for a period of time;  1. Set all accumulated variables $ADT_{i}$ to initial value 0;  2. for (each position corresponding to a regular time interval on the trajectory) 3. for (each triangular unit $DT_{i}$) 4. { 5. if (APIT($DT_{i}$) == outside) $ADT_{i}$ maintains unchanged; 6. if (APIT($DT_{i}$) == inside) $ADT_{i}$ incremented by 1; 7. } 8. Find the sequential triangular regions with the adjacent maximal accumulated values.

The symbol ∘ represent a series of test positions (see Figure 6). The algorithm shows how the aggregated variables reflect the positional information in both the temporal and spatial domains. The continuous APIT tests reveal the time dimension information and Delaunay triangulation reveals the spatial dimension information. The value of the accumulated variables depends on the length of the trajectory separated by triangular regions (the length will largely determine the number of APIT tests).

### 3.3. Boundary Detection Based on Time Series

**Figure 7:**
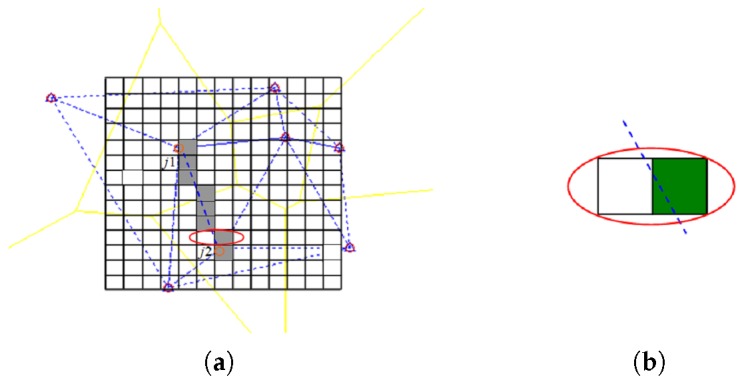
Boundary fingerprint table: (**a**) Boundary j1j2; (**b**) Select grid.

A classic fingerprinting algorithm consists of two phases: offline and online. In the first phase, the experimental site is divided into 0.5 m × 0.5 m square grids and *RSSI* measurements are collected at each intersection. In the online phase, the *RSSI* vector information of a moving object will be detected and recorded. Then, the received *RSSI* vector will be compared with the fingerprint map table to determine the position of the moving object. Finally, the trajectory is formed by mining the position information. In our scheme, to detect the position related to the peak value on the boundary, a boundary fingerprint table is organized independently and indexed by the boundary information. When a segment of a boundary belongs to two adjacent square grids, the square grid with the higher ratio is selected as the boundary fingerprint (Boundary j1j2 is shown in Figure 7a, the green grid is selected in Figure 7b. Figure 7b is an enlargement of the red ellipse in Figure 7a. The boundary fingerprint format is as follows:

(10) $$\begin{array}{c} ((j1,j2),(x,y),RSSI\;vector\;mean), \end{array}$$

where j1 and j2 are the endpoints of boundary j1j2, *x* and *y* are coordinates of the square grid, and RSSI vector mean is the corresponding position fingerprint. For the purpose of tractability, j1≤j2 is the requirement.

**Figure 8:**
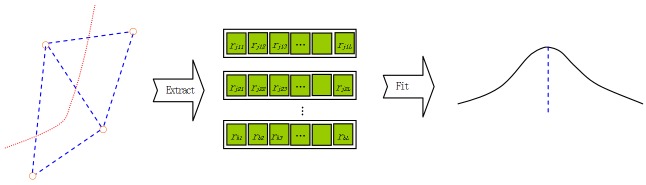
Boundary detecting based on time series.

Unlike the existing fingerprinting model, we can determine the boundary on which the unknown node resides using the peak value before matching the fingerprint. To reduce the impact of noise, we use a moving least square (MLS) method to process the boundary time series $R_{b}$ (see Figure 8). The discrete *RSSI* information is converted into a continuous curve in the temporal dimension. Then, the change tendency and peak value of the curve will be detected. MLS is a segmented least-square fitting method that ensures the curve will be smooth. The MLS formulas are as follows:

(11) $$\begin{array}{c} \;linear\;base\;\;\;\;\;\;\;\;\;\;P\left(x\right)={\left[1,x\right]}^{T}, \end{array}$$

(12) $$\begin{array}{c} \;coefficient\;vector\;\;\;\;\;\;\;\;\;\;a\left(x\right)=A^{-1}\left(x\right)B\left(x\right)y, \end{array}$$

where

(13) $$\begin{array}{c} A\left(x\right)=\sum_{j=1}^{n}w\left(x-x_{j}\right)p\left(x_{j}\right)p^{T}\left(x_{j}\right), \end{array}$$

and *n* represents the number of nodes within the range of influence. Then,

(14) $$\begin{array}{c} B\left(x\right)=[w\left(x-x_{1}\right)p\left(x_{1}\right),w\left(x-x_{2}\right)p\left(x_{2}\right),\ldots ,w\left(x-x_{n}\right)p\left(x_{n}\right)] \end{array}$$

and

(15) $$\begin{array}{c} y^{T}=\left[y_{1},y_{2},\ldots ,y_{n}\right], \end{array}$$

and the fitting function is

(16) $$\begin{array}{c} f\left(x\right)=\sum_{i=1}^{m}a_{i}\left(x\right)p_{i}\left(x\right), \end{array}$$

where *m* represents the number of terms in the linear base:

(17) $$\begin{array}{c} w\left(\gamma \right)=\left\{\begin{array}{cc} 2/3-4\gamma^{2}+4\gamma^{3}, & (\gamma \leq 1/2),\\ 4/3-4\gamma +4\gamma^{2}-4\gamma^{3}/3, & (1/2<\gamma \leq 1),\\ 0, & (\gamma >1), \end{array}\right. \end{array}$$

and

(18) $$\begin{array}{c} \gamma =\left(x-x_{i}\right)/\gamma_{max}, \end{array}$$

where $\gamma_{max}$ represents the length of the range of influence. The weighted sum of several of the surrounding primitive *RSSI* measurements substitutes for the primitive *RSSI* measurement. The cubic spline function w(γ) is selected as the weight function. The weight of each primitive data point is different. The smaller the distance between the primitive point and the substitutive point, the larger the corresponding weight (see Equations (16) and (17)). The curve fitting accuracy depends on the rank number of the base function P(x). Based on field experiments, a linear base is selected.

After the peak value is detected, we can obtain the corresponding time instant (in Figure 8, the blue perpendicular dotted line indicates the time instant). Then, the fingerprint at this time instant can be matched to the boundary fingerprint table. The start and end points of the segmented trajectory are determined. Compared with the conventional fingerprint matching algorithm, the boundary information is highly accurate at the given time instant. This conclusion has been verified both theoretically and through practical application.

The pseudocode for calculating the position at the time instant related to the peak value is as Algorithm 2:
**Algorithm 2** Calculating the Position at the Time Instant Related to the Peak ValueInput: boundary fingerprint table, adjacent triangular regions, boundary *RSSI* time series $R_{b}$; Output: start and end points of the segmented trajectory;  1. {  2. Fit boundary *RSSI* time series $R_{b}$ by MLS; 3. Find the peak value; 4. Determine the start and end points of the segmented trajectory. 5. }

### 3.4. Dynamic Time-Warping Position-Fingerprint-Matching Algorithm with Constraints

**Figure 9:**
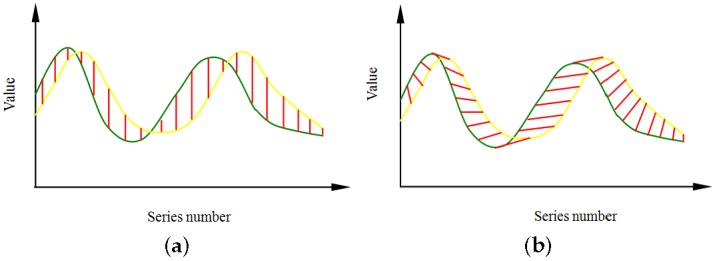
Dynamic time warping principle: (**a**) Euclidean distance; (**b**) DTW (Dynamic time warping) distance.

Dynamic time warping (DTW) is a kind of nonlinear warping technology that combines warping in the time dimension with distance measurements [23]. DTW uses nonlinear warping technology to determine the maximal overlap between two paths, essentially removing the time difference. As shown in Figure 9a, the Euclidean distance measurement requires aligning the data in the time dimension; in DTW, the distance measurement eliminates the time difference (see Figure 9b). DTW can achieve minimal distortion in terms of change tendency measurement [23]. Moreover, it can process two time series of different lengths and is a robust approach.

**Figure 10:**
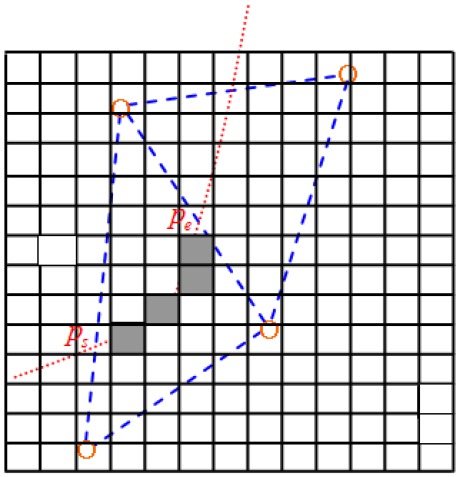
Constrained position fingerprint matching.

Position fingerprints corresponding to a segment of trajectory can be processed as a time series. As shown in Figure 10, the two endpoints of the segmented trajectory within a triangular region are on different boundaries, respectively. We search for the most similar candidate trajectory within the triangular region for the time series detected. The DTW distance measurement is defined as follows:

(19) $$\begin{array}{c} D_{DTW}\left(U,W\right)=D_{base}\left(u_{1},w_{1}\right)+min\left\{\begin{array}{c} D_{DTW}\left(U,W\left[2:-\right]\right),\\ D_{DTW}\left(U\left[2:-\right],W\right),\\ D_{DTW}\left(U\left[2:-\right],W\left[2:-\right]\right), \end{array}\right. \end{array}$$

where $U=(u_{1},u_{2},\ldots ,u_{n1})$ represents a time series in the online phase, $W=(w_{1},w_{2},\ldots ,w_{n2})$ represents a candidate time series in the offline phase, and n1 and n2 are the lengths of *U* and *W*, respectively, and

(20) $$\begin{array}{c} D_{base}\left(u_{1},w_{1}\right)={∥u_{1}-w_{1}∥}^{2}, \end{array}$$

where $D_{base}\left(u_{1},w_{1}\right)$ represents the Euclidean distance between position fingerprints $u_{1}$ and $w_{1}$. The recursive definition of $D_{DTW}$ can measure the similarity between two time series with different lengths. Equation (19) shows that the DTW algorithm can be implemented with dynamic programming. The three conditions that must be satisfied by each candidate trajectory time series are as follows:

1. The start point is $p_{s}$, and the end point is $p_{e}$, where $p_{s}$ and $p_{e}$ represent the heuristic information on the boundary.
2. The candidate trajectory must reside entirely within the triangular region.
3. The candidate trajectory must be continuous, which means that the trajectory must be composed of a series of position fingerprints related to adjacent square grid units (see Figure 10).

The length of a candidate trajectory *W* is not necessarily equal to that of *U*. We introduce the DTW algorithm to match position fingerprints with different lengths. The most similar candidate trajectory is regarded as the tracked trajectory.

Compared with traditional fingerprint matching, on one hand, this approach mainly considers the change tendency of a trajectory, while on the other hand, the continuous position fingerprint in the time dimension must also be continuous in the space dimension.

The pseudocode for the dynamic time-warping position-fingerprint-matching algorithm is as Algorithm 3:
**Algorithm 3** Dynamic Time-Warping Position-Fingerprint-Matching AlgorithmInput: fingerprint time series, triangular region, start point, end point, fingerprint map table; Output: the most similar candidate trajectory within the triangular region;  1. {  2. Fit fingerprint time series by MLS; 3. For (each candidate trajectory satisfying the three conditions mentioned above) 4. Calculate $D_{DTW}$ between the candidate trajectory and the fitted fingerprint time series; 5. Find the minimal $D_{DTW}$, which denotes the most similar candidate trajectory. 6. }

## 4. TTDH Performance Evaluation

**Figure 11:**
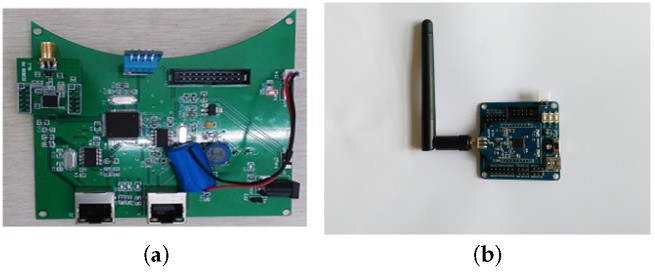
Nodes: (**a**) Reference node; (**b**) Unknown node.

**Figure 12:**
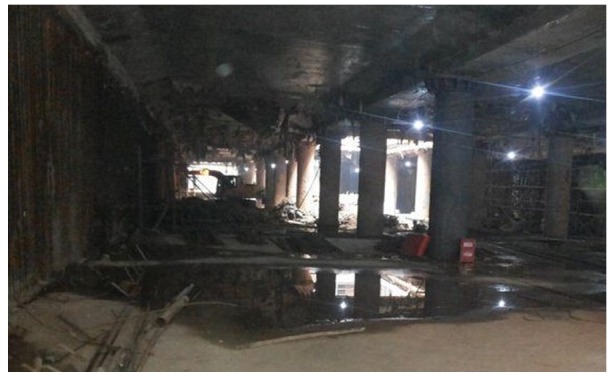
Deep foundation pit of a metro station.

To verify the performance of our TTDH trajectory tracking scheme, this section describes a series of field experiments in which we used a CC2530 transceiver chip. A reference node and an unknown node are shown in Figure 11. The indoor area is a deep foundation pit for a metro station whose size is 50 m × 30 m (Figure 12). All the reference nodes are randomly deployed on the roof of the pit. Workers wearing helmets and construction machinery move about in the region as needed. Each unknown node is attached to a helmet or resides on a transporter. The trajectory of a worker or a transporter is formed by tracking the corresponding unknown nodes. There are no intervening walls in the area.

### 4.1. TTDH Framework

The steps of the TTDH scheme are as follows:

Step 1. Perform Delaunay triangulation based on the positional information of all reference nodes.
Step 2. Collect position fingerprints in square grid units and form a fingerprint map table.
Step 3. Organize a boundary fingerprint table and create an index on this table with the boundary information.
Step 4. Perform continuous Delaunay triangulation inside/outside decisions to obtain the sequential triangular regions.
Step 5. Determine the start and end points of a segmented trajectory.
Step 6. Execute the dynamic time-warping position-fingerprint-matching algorithm to form a trajectory segment.

The first three steps are initialization steps. Then, the remaining steps are repeated with tracking processes. We assume that the reference nodes have not been moved. If a reference node is moved, we start all the steps over again.

### 4.2. Fingerprint Collection

The TTDH scheme requires a fingerprint map table and a boundary fingerprint table. Considering the computing capability and storage capacity of a wireless node, we perform this computing work at a central computer. That is, fingerprints are collected and stored by the central computer in the offline phase. Then, real-time *RSSI* measurements received by an unknown node are uploaded to this computer and processed during the online phase.

### 4.3. Key Data Structure Setting

The APIT tests are performed continuously by exchanging information between a monitored node and its neighbors. A monitored node is allocated a group of accumulated variables whose number is equal to the total number of triangular regions.

It must be determined whether a point is inside/outside a triangular region. The exchanged information consists only of the ID numbers of the reference nodes, the ID numbers of the neighboring nodes and the corresponding *RSSI*, as shown in Table 1. Each monitored node’s information is transmitted to the central computer and merged (Table 2).

By searching the columns in the merged information table, if a column exists in which all members are larger/smaller than those of the monitored node, we can assume that the point is outside the region. If no such column is found, we can assume that the point is inside. Therefore, the decision regarding any triangular region can be made as described above and all triangular regions within the node’s communication radius can be determined in the same way.

In a fixed time interval, a series of APIT tests will be performed, and the results will be aggregated. For trajectory tracking, the continuous APIT tests based on Delaunay triangulation reflect a spatiotemporal correlation. A single inside/outside decision error can be masked by the aggregation mechanism.

### 4.4. Boundary Detection

**Figure 13:**
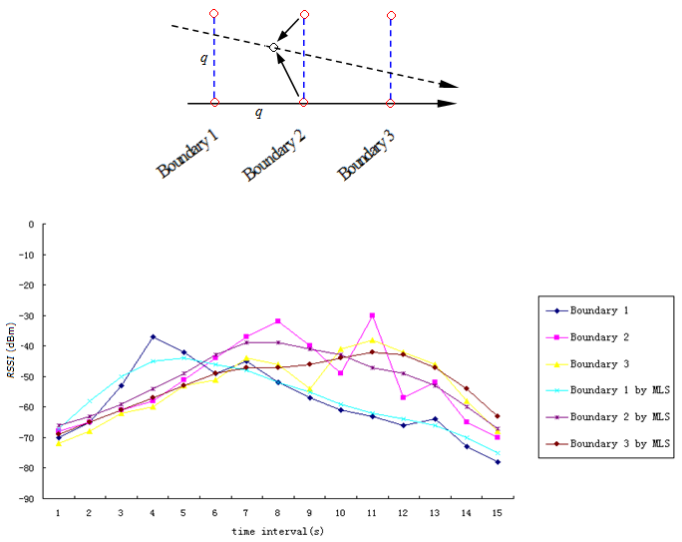
Boundary detecting.

We performed independent field experiments to detect the peak value on the boundary. As shown in Figure 13, the reference nodes are deployed in square grid units where the length of each side, *q*, is equal to 7 m.

Overall, the experimental results show that the boundary time series monotonically decreases as the distance increases. There are some exceptions in the discrete *RSSI* measurements (e.g., the peak value on boundary 2). A boundary time series is fitted into a smooth curve, and by using MLS, we can effectively eliminate the impact from such exceptions. In general, our scheme can obviously detect the peak value on each boundary. The three perpendicular dotted lines indicate the time instants when the monitored node passes through each boundary. Note that a small bias exists between the second perpendicular dotted line and the real position. The reasons causing this bias are as follows:

1. A reference node communicates with an unknown node at regular time intervals. It is difficult to guarantee that the communication occurs exactly on the boundary.
2. The uncertainty of *RSSI* measurements introduces error.

Fortunately, the experimental results show that the probability that the bias is less than 1 s in a 0.5 s time interval is above 93%, which translates to an error distance of less than 1.3 m.

### 4.5. Triangular Region Detection

**Figure 14:**
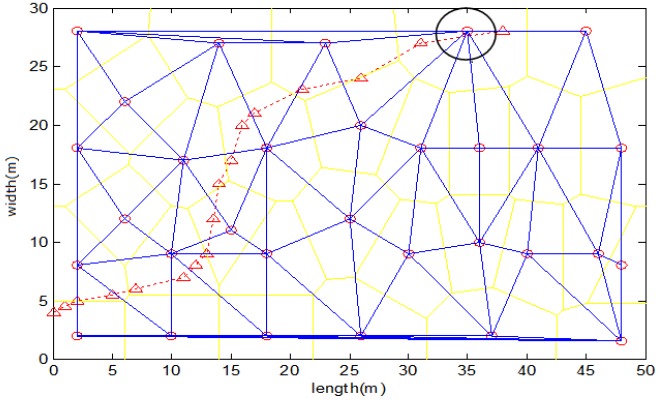
Region detecting based on Delaunay triangulation.

There are 31 reference nodes in the study area. The corresponding Voronoi diagram and Delaunay triangulation are shown in Figure 14, where the red symbol ∘ represents a reference node and the yellow line segments constitute a Voronoi diagram. In the Delaunay triangulation, a blue line segment is a boundary. The heuristic information means the triangular regions and boundaries crossed by the trajectory. The red dotted line denotes a test trajectory.

**Figure 15:**
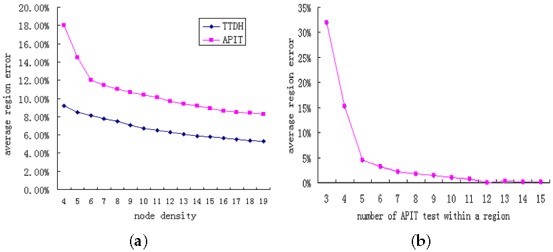
TTDH average region error: (**a**) Under varying node density; (**b**) Under varying region area.

Figure 15a shows the average triangular-region error percentage as a function of node density, while Figure 15b shows this error percentage as a function of the number of APIT tests within a triangular area.

1. The APIT test employs neighbor information to emulate the movement of a monitored node in different directions. The higher the node density is, the more accurate the triangular region detection will be. When the node density is greater than 5, the average region error percentage falls to 8.5% accuracy improvement compared with classical APIT.
2. Our scheme aggregates the results of discrete APIT tests to decide which triangular region has been crossed. The larger the triangular area is, the more APIT tests need to be performed. The greater the number of APIT tests is, the more accurate the triangular region detection will be. When the number of APIT tests within a triangular region is greater than 5, the region error percentage falls to 4.5%.
3. The impact of the movement speed of a monitored node is the same as the impact of the size of a triangular area, which determines the number of APIT tests within a triangular region. For an object at high speed, we can reduce the time interval discussed above to improve the accuracy.
4. Because the number of APIT tests is a key factor in the accuracy of triangular region detection, when sharp or narrow regions are crossed, the triangular region is not easily detected. As shown in Figure 14, the circled region in the upper right corner is difficult to detect. Sometimes, this phenomenon can be corrected by means of the continuity of a moving trajectory and information from adjacent regions (e.g., when two large regions have definitely been crossed, the short, sharp region between them must also have been crossed).

### 4.6. Forming A Trajectory by Means of the Dynamic Time-Warping Position-Fingerprint-Matching Algorithm

Based on the triangular region and boundary information, trajectory tracking becomes a segmented position-fingerprint-matching procedure. The corresponding positions on the boundary are used as the start and end points of the segmented trajectory. The average error distance (the Euclidean distance between the real position and the estimated position) of the tracked trajectory will fall to less than 1.5 m. A sliding window of 10 *RSSI* samples was mentioned in RADAR. Our TTDH scheme is essentially a sliding window method too: the difference is that one uses a standardized window size and the other uses an adjustable size. In RADAR, the continuous movement is split into a series of regular sliding windows mechanically, while, in our scheme, the sliding window is combined with the reference nodes deployment and the monitored node movement.

The start and end points are on the common side of two adjacent triangular regions; therefore, the end point of the former region is the start point of the latter region. The DTW algorithm matches data from a sequence of fingerprints rather than one. A segment of the trajectory is formed independently, and each peak value with high accuracy is used as a calibrated point. The error does not accumulate among the triangular regions. These factors make the tracked trajectory smooth and accurate.

Field experiments show that our method performs better than RADAR, which is a typical method based on position fingerprint. Even when the *RSSI* measurements include a large amount of signal noise, the trajectory tracked by our method is more accurate due to the region and boundary constraints.

### 4.7. Impact of Multipath Effect and Deployment of Reference Nodes

In the fields of location and trajectory tracking, multipath effect leads to time delay, which has a serious effect on TOA (Time of Arrival) localization algorithm [5]. In this paper, we use the algorithm based on *RSSI* position fingerprint, and the signal strength fluctuation caused by multipath fading is the main influence. In order to reduce the impact of discrete *RSSI* fluctuation, the following measures have been adopted:

1. Due to the presence of multipath effect, *RSSI* will fluctuate and result in a possible error in the single APIT test. As previously mentioned, the error rate of single APIT test is relatively low when the node density is greater than 6. The results of discrete APIT tests are aggregated to decide which triangular region has been crossed. The accuracy of triangular region detection is mainly determined by the number of APIT tests. When the number of APIT tests within a triangular region is greater than 5, the region error percentage can reach 95.5%. The aggregation mechanism can effectively mask a single inside/outside decision error caused mainly by multipath effect. On average, node density greater than 6 and triangular regions with relatively large areas are required in TTDH.
2. As shown in Figure 13, there are some exceptions in the discrete *RSSI* measurements caused mainly by the multipath effect. A boundary time series is fitted into a smooth curve by using MLS. The change tendency of boundary time series $R_{b}$ is more easily detected than the direct localization. TTDH can effectively eliminate the impact from such exceptions. The experimental results show that the probability of a bias occurring of less than 1 s in 0.5 s time interval is above 93%, which means an error distance of less than 1.3 m.
3. Fingerprints are measured in the actual environment, and environmental factors (e.g., multipath effect) have been considered. The track is formed by using a DTW (dynamic-time-warping position-fingerprint-matching algorithm with heuristic information constraints) algorithm. The Rayleigh fading model [7] and the Rician distribution model [7] are not used, which are mainly used in range based localization system.
4. When a Delaunay triangle is too large, the real-time performance will be bad due to TTDH forming a segment of trajectory as a unit of triangular region. Therefore, the Delaunay Triangulation related to the positions of all reference nodes should try to be balanced in size and shape and avoid forming sharp or narrow regions.

## 5. Conclusions

In this paper, we described the design and development of a novel indoor trajectory tracking scheme called TTDH. In this method, Delaunay triangulation is performed based on reference nodes as spatial information, and the peak value of time series is regarded as temporal information. TTDH makes the best of the temporal information and spatial information in WSNs to mine the heuristic information. This heuristic information includes the triangular region in which an unknown node is located and the boundary on which the unknown node resides at the peak value of the corresponding time series. Then, TTDH forms a segment of trajectory of the monitored object using a dynamic-time-warping-position-fingerprint-matching algorithm with constraints. In this procedure, the aggregation mechanism of discrete APIT tests can effectively improve the accuracy of triangular region detection and the boundary information can be detected with high accuracy.

Field experiments show that TTDH achieves good trajectory accuracy and smoothness. The average error distance of this trajectory tracking method can be reduced to less than 1.5 m, and the error does not accumulate among the triangular regions. In summary, TTDH combines a fingerprint matching method with a range-free localization method, and its good performance stems from making full use of heuristic information.

## Figures and Tables

**Table 1:** Exchanged information table.

Reference Node ID	Unknown Node ID
A	$RSSI_{A}$
B	$RSSI_{B}$
C	$RSSI_{C}$

**Table 2:** Merged information table.

Reference Node ID	Unknown Node Self-ID	Unknown Node ID${}_{1}$	⋯	Unknown Node ID${}_{m}$
A	$RSSI_{A}$	$RSSI_{A}$	⋯	$RSSI_{A}$
B	$RSSI_{B}$	$RSSI_{B}$	⋯	$RSSI_{B}$
C	$RSSI_{C}$	$RSSI_{C}$	⋯	$RSSI_{C}$
